# Associations of eating context with dietary quality, satiety, and postprandial blood glucose in free-living Singaporean adults during 9 days of intensive digital phenotyping

**DOI:** 10.1186/s12966-026-01903-2

**Published:** 2026-03-18

**Authors:** Leah Turton, Jiali Yao, Salome A. Rebello, Xueling Sim, Falk Müller-Riemenschneider, Rob M. van Dam

**Affiliations:** 1 Department of Exercise and Nutrition Sciences, Milken Institute School of Public Health, The George Washington University, 950 New Hampshire Ave NW #2, Washington, DC 20052 USA; 2 Saw Swee Hock School of Public Health, National University of Singapore, 12 Science Drive 2, Singapore, 117549 Singapore; 3 Berlin Institute of Health, Charité-Universitätsmedizin Berlin, Berlin, Germany; 4 Harvard T.H. Chan School of Public Health, Boston, Massachusetts USA

## Abstract

**Background:**

Eating behaviors are shaped by contextual factors such as where people eat, who they eat with, and their activities and mood during meals. However, data on these determinants of food choice in Asian populations are limited. We examined how eating context is associated with dietary quality, satiety, and postprandial blood glucose levels in an urban Asian population.

**Methods:**

We analyzed data from up to 1291 Singapore residents aged 21–69 years (20,629 meals) in the Continuous Observations of Behavioral Risk Factors in Asia (COBRA) study. Over nine consecutive days, participants completed smartphone-based ecological momentary assessments six times per day, reporting meal composition, location, companions, concurrent activities, and premeal hunger, tiredness, stress, and happiness. We calculated diet quality scores for each meal (range 0–10). Masked continuous glucose monitors recorded interstitial glucose every 15 min, from which the 2-hour postprandial glucose was derived using incremental area under the curve. We estimated associations using generalized estimating equations, adjusting for sociodemographic, lifestyle, clinical, and other contextual factors.

**Results:**

Meals were consumed at home (60%), hawker centers (local open-air food courts) (14%), the workplace (11%), other restaurants (9%), fast-food restaurants (2%), or other locations. Compared with home meals, diet quality was significantly lower at all out-of-home locations, particularly at fast-food restaurants (β: -0.70; 95% CI: -0.82, -0.57) and hawker centers (β: -0.56; CI: -0.63, -0.48). Postprandial glucose was higher after meals at hawker centers (β: 30.11 mmol/L*minute; CI: 20.11, 40.11) and the workplace (β: 17.48; CI: 4.34, 30.62) than at home. Eating with friends was associated with lower postprandial glucose than eating alone. Higher premeal happiness was associated with modestly higher diet quality, whereas greater premeal hunger was associated with higher postprandial glucose.

**Conclusions:**

In this urban Asian setting, eating location was a key determinant of meal quality and postprandial glycemic response. Hawker centers and fast-food restaurants were associated with worse diet quality, and hawker centers and workplace venues with higher postprandial glucose. Interventions that support home-prepared meals and promote healthier food options when eating away from home may improve cardiometabolic health.

**Supplementary Information:**

The online version contains supplementary material available at 10.1186/s12966-026-01903-2.

## Introduction

Dietary risk factors significantly contribute to the global disease burden. They were linked with 3.4 million deaths in women and 4.47 million deaths among men in 2019 [1]. Diets high in sodium, processed meats, and sugar-sweetened beverages and low in nuts/seeds, fruits, vegetables, and seafoods are associated with a higher risk of chronic diseases, including type 2 diabetes and cardiovascular disease [2]. The sphere of influence on an individual’s dietary behaviors is complex. Influences on diet behaviors include the eating environment, mental health or stress levels, and the use of digital devices or social interactions during mealtimes [3–13]. Singapore, like many nations, has experienced an increased prevalence of cardiometabolic conditions such as type 2 diabetes, hypertension, and hypercholesterolemia in recent decades [14]. Given the eating context and dietary patterns in Singapore, findings on determinants of dietary behaviors may inform the development of nutrition-related health interventions across urban Asian settings [15–17].

The Social Ecological Framework provides context for the determinants of health at the individual, interpersonal or relationship, community, and societal levels [18, 19]. To explore multi-level factors influencing health behaviors in real time, researchers are increasingly employing Ecological Momentary Assessment (EMA) applications [20]. EMA allows the collection of real-time data in the “natural environment”, thus moving beyond historical, recalled data from participants [20]. In nutrition research, the use of EMA holds additional promise for providing a behavioral and physiological context for dietary behaviors within distinct timeframes [20, 21]. This technology is an effective data-collection method in behavioral nutrition research across diverse populations, including individuals with type 2 diabetes and binge-eating disorders, as well as healthy young adults [22–25]. However, many EMA studies were small in sample size, did not assess postprandial blood glucose levels, or were conducted in populations with specific chronic diseases [22–25].

The aim of this study was to examine how the eating context influences meal quality, postprandial fullness, and postprandial blood glucose levels. We therefore conducted a 9-day EMA-based study with continuous glucose monitoring in an adult Singapore population under free-living conditions. We examined multiple eating context factors, including meal companions, meal location, concurrent activities during meals, and premeal psychophysiological states. We hypothesized that these eating context factors were associated with an individual’s dietary quality and blood glucose control.

## Methods

### Study design and participants

The Continuous Observations of Behavioral Risk Factors in Asia (COBRA) is an intensive longitudinal observational study conducted between May 2021 and August 2024. COBRA was nested within the Singapore Multi-Ethnic Cohort (MEC), a population-based cohort of community-dwelling adults [26]. MEC participants were recruited through public outreach and referrals, with ongoing enrolment to enhance the representativeness of the general Singapore population, except for targeted oversampling of Malays and Indians (the ethnic minority groups). Between May 2021 and August 2024, eligible MEC participants (*N* = 2304) were invited to participate in COBRA, and 1304 agreed to participate and completed the study (response rate: 57%). This sample size was maximized as permitted by the logistical and financial resources, the study requirements, and the study timeline. The detailed study protocol was published elsewhere [27, 28]. Briefly, COBRA recruited Singapore males and females aged 21–69 years of Chinese, Malay, or Indian ethnicity. Additional eligibility criteria were having a smartphone with a data plan and the ability to read English, use smartphone apps, and walk independently. The exclusion criteria included having an allergy to medical-grade adhesive, bleeding disorders, severe mental health conditions, or a history of cardiovascular disease, diabetes, cancer, kidney failure, or thyroid disorders. All participants provided informed consent. Ethical approval was obtained from the Institutional Review Board of the National University of Singapore.

Participants first had a baseline visit at the National University of Singapore, followed by nine days of continuous free-living observations. The baseline visit included a structured questionnaire interview to collect participants’ sociodemographic, medical, and lifestyle information. The baseline visit also involved a standardized physical examination with blood samples collected for biochemical analyses, including HbA1c and fasting plasma glucose. Notably, although we did not plan to recruit individuals with diabetes, 55 participants were identified as having the condition, most of whom were newly diagnosed based on physical examination results (HbA1c ≥ 6.5% or fasting plasma glucose ≥ 7 mmol/l). Anthropometric measures were conducted at baseline by trained technicians. Height was measured without shoes on a portable stadiometer (SECA 200 series, Germany) with the head in the Frankfurt Plane position. Weight was measured on a digital scale (SECA 700 series, Germany). Body mass index (BMI) was calculated as weight divided by height squared (kg/m^2^).

At the baseline visit, a study app (Avicenna; Avicenna Research Inc) was also installed in participants’ smartphones, and a masked continuous glucose monitoring (CGM) device (Freestyle Libre Pro iQ; Abbott Diabetes Care) was fitted on the participant’s upper non-dominant arm. During the free-living days, the app delivered six EMA surveys per day at random times within six pre-defined time windows (i.e., 8:00–9:30, 10:30 − 12:00, 13:00–14:30, 15:30 − 17:00, 18:00–19:30, and 20:30 − 21:30 h), while the CGM device automatically collected the interstitial glucose level every 15 min.

### Assessment of meal intake, quality, and context

Participants’ meal composition, meal consumption location, meal companion, concurrent activity during the meal, and momentary psychophysiological states were captured through EMA surveys.

On each day, the first EMA survey captured participants’ food intake during the period after the last EMA survey and before sleep yesterday (applicable to free-living days 2–9) and during the period from waking up that morning to the first EMA prompt. The subsequent five EMA surveys captured participants’ food intake since the previous EMA survey. Food intake and eating time (HH: MM format) for meal events were reported. Meals consumed during 05:00–24:00 of the day were included for analysis. To reduce participant burden and increase survey adherence, we did not ask participants to provide detailed food information (e.g., portion sizes). Instead, participants selected applicable items from predefined, Singapore-specific lists of food groups, using check-all-that-apply questions that allowed multiple responses. Specifically, participants reported their meal intake by responding to the question ‘What foods were part of your meal?’. The pre-defined meal food groups included refined grains (e.g., white rice, noodles, pasta, bread, or cereal), whole grains (e.g., brown or wholegrain rice, noodles, pasta, bread, or cereal), seafood, chicken, red meat (e.g., beef, pork, mutton, lamb), eggs, dairy products (e.g., milk, yogurt, cheese), soy food (e.g., tofu and tempeh), beans, peas, nuts, seeds (e.g., peanuts, dahl, sambar), vegetables, fruit, deep fried foods (e.g., puffs, samosas, French fries, fried chicken), and sweet desserts. Other food items that participants could not directly match to the predefined food groups were recorded in free-text. These free-text food entries were independently examined by two researchers familiar with local food options and mapped to or merged with the closest food groups in the predefined list; disagreements were resolved by consensus or by a third researcher.

The meal intake data were used to derive a diet quality score, adapted from the Dietary Approach to Stop Hypertension (DASH) diet score [29]. For each meal, a value of 1 was assigned for consumption of each of the following food groups: whole grains, seafood, legumes (i.e., beans, peas, lentils, soy products) or nuts, vegetables, fruit, and dairy. Furthermore, a value of 1 was assigned for non-consumption of the following ‘unhealthy’ food groups: refined grains, red meat, deep-fried foods, and sweet desserts. The quality score was calculated as the sum of these ten food group values (range: 0–10).

For each meal event, participants also responded to questions on postmeal fullness (‘How full did you feel after eating your meal?’; Likert scale with value 1 = still hungry and 6 = extremely full; single-choice response), meal location (‘Where did you eat your meal?’; single-choice response), meal companion (‘Who were you with?’; multiple-choice response; check-all-that-apply), and activities during the meal (‘What did you do while you were eating?’; multiple-choice response; check-all-that-apply) [27].

During each EMA survey, participants’ momentary psychophysiological states were captured using the question ‘How stressed/hungry/tired/happy do you feel right now?’ with a visual analog scale from 0 (= not at all) to 6 (= very much) [27]. The app automatically recorded timestamps for the psychophysiological states (different from participant-reported mealtime). Data of the latest psychophysiological states within 2 h before a meal were available for 57% of the meals and were extracted to define the premeal psychophysiological states.

### Assessment of postprandial glucose response

We used time-matched CGM data to derive the 2-hour incremental area under the curve (iAUC) of postprandial glucose levels using the trapezoidal rule [30]. The derivation was restricted to the 61% meals with complete CGM data over the 2-h postprandial period and at least 2 h of fasting both before and after the meal.

### Statistical analysis

Participant and meal characteristics, overall and stratified, were summarized using descriptive statistics. To assess whether meal companion and activity during the meal varied by meal location, we conducted Wald tests comparing null generalized estimating equation (GEE) models with GEE models including meal location as the only independent variable. Each dependent variable represented a specific meal companion or activity during the meal. This approach was chosen to account for (1) the clustered nature of the repeated meal data and (2) the non-mutually exclusive nature of response options in the check-all-that-apply questions (applicable for meal composition, meal companion, activity during the meal). Similarly, this approach was used to examine whether diet composition, diet quality, postprandial satiety, and postprandial glucose response varied by meal location, meal companion, and activity during the meal. In addition, crude correlation coefficients were computed between the premeal psychophysiological states and meal composition, diet quality, postprandial satiety, and postprandial glucose response. Given the clustered nature of the data, the statistical significance was determined based on the correlation distribution estimated from 10,000 bootstrapping samples of the original meal data. Given the exploratory nature of our study, we did not adjust p-values for multiple testing.

We conducted multivariable GEE models to estimate adjusted associations of eating context factors (meal location, meal companion, activity during the meal, and premeal psychophysiological states; independent variables) with diet quality, postprandial satiety, and postprandial glucose response (dependent variables). Model covariates included participant age (years), biological sex (male and female), ethnicity (Chinese, Malay, and Indian), glycemic status (normoglycemia, prediabetes [without diabetes but HbA1c ≥ 5.7% or fasting plasma glucose ≥ 5.6 mmol/l], and diabetes), BMI (kg/m^2^), cigarette smoking (yes/no), heavy alcohol drinking (yes/no having at least five servings of alcohol in a single drinking session in males or at least four servings in females), the highest education attainment (below A-level, A-level or equivalent, and university or above), working status (yes/no current working), marital status (yes/no married or living with a partner), day of week (weekday/weekend), and mealtime (around breakfast: 05:00–11:00, lunch: 11:00–17:00, and dinner: 17:00–24:00 h). The multivariable GEE models were simultaneously adjusted for meal location, meal companion, and activity during the meal. Because premeal psychological state data were available for only 57% of meals, our primary models examining associations with meal location, meal companion, and activity during the meal did not adjust for psychophysiological states to avoid reducing the sample size and compromising statistical power. Sensitivity models were performed that simultaneously adjusted for all eating context factors, including psychophysiological states. The multivariable GEE models were conducted using with robust sandwich standard error estimators and an independent working correlation structure. We also conducted sensitivity models using an exchangeable working correlation structure and an autoregressive working correlation structure.

All analyses were conducted in R (version 4.4.2). Hypothesis tests were two-sided, with p-values < 0.05 considered statistically significant. GEE models were performed using the R package ‘geepack’ (version 1.3.12), with meals as observations and participants as clusters. The identity link function was used for models with numeric dependent variables, and the logit link function for models with binary dependent variables. The R package ‘marginaleffects’ was used to compute adjusted marginal estimates from GEE models. Variance inflation factors were used to assess multicollinearity in the multivariable models through the R package ‘car’ (version 3.1-3).

## Results

### Characteristics of participants and meals

Of the 1,304 recruited participants, 1291 participants with EMA-based data for more than one meal were included in this study (Table 1). One participant without baseline assessment and 12 participants without EMA-based meal data were excluded. The included 1291 participants responded to 63,408 EMA surveys (91% of the scheduled). These participants contributed 20,629 meals spread across the day for analysis, of which 11,783 (57%) meals had time-matched pre-meal psychological state data. The mean participant age was 41.0 years (standard deviation: 14.1), and the mean BMI was 24.9 kg/m² (standard deviation: 5.6). Most participants were female (62%) and of Chinese ethnicity (64%). The other ethnicities were Malay (21%) and Indian (15%). Only a small proportion of the population was smokers (13%) or heavy alcohol consumers (8%). Most participants had normal glycemia (70%), and about 30% had prediabetes (25%) or diabetes (4%). Among all participants, 971 participants provided 12,622 meals with postprandial glucose response data, and 7161 meals with both postprandial glucose response and pre-meal psychological state data. The characteristics of these participants and their meals were similar to those of all participants.

**Table 1:** Participant and meal characteristics for all participants and those with postprandial glucose data

	All participants	Participants with postprandial glucose data
Participant characteristics		
Number of participants	1291	971
Age (years)	41.0 (14.1)	40.7 (13.8)
Female	804 (62%)	601 (62%)
Ethnicity, Chinese	821 (64%)	622 (64%)
Malay	271 (21%)	202 (21%)
Indian	199 (15%)	147 (15%)
Education, Secondary or below	178 (14%)	124 (13%)
A-level	419 (32%)	298 (31%)
University or above	694 (54%)	549 (57%)
Cigarette smoking, current	171 (13%)	133 (14%)
Heavy alcohol consumption	97 (8%)	76 (8%)
Currently working	1012 (78%)	770 (79%)
Married or living with a partner	656 (51%)	488 (50%)
Glycemia, normal	908 (70%)	686 (71%)
Prediabetes	329 (25%)	240 (25%)
Diabetes	54 (4%)	45 (5%)
Body mass index (kg/m^2^)	24.9 (5.6)	25.0 (5.7)
Fasting glucose (mmol/L)	5.1 (0.9)	5.2 (1.0)
HbA1c (%)	5.5 (0.6)	5.5 (0.6)
Meal characteristics		
Number of meals	20,629	12,622
Day of week: weekdays	14,221 (69%)	8796 (70%)
Mealtime, 05:00–11:00 h	5031 (24%)	3252 (26%)2
11:00–17:00 h	7769 (38%)	4806 (38%)
17:00–24:00 h	7829 (38%)	4564 (36%)
Stress, premeal rating*	1.57 (1.37)	1.55 (1.38)
Hunger, premeal rating*	2.25 (1.52)	2.27 (1.52)
Tiredness, premeal rating*	2.25 (1.48)	2.24 (1.48)
Happiness, premeal rating*	3.63 (1.36)	3.63 (1.37)
Diet quality score**	4.13 (1.38)	4.13 (1.38)
Postprandial fullness rating§	3.80 (1.04)	3.80 (1.05)
Postprandial glucose iAUC (mmol//L*minute)	143.28 (137.52)	143.28 (137.52)

Date in *N*, *N* (%), or Mean (SD); *Score ranges from 0 (‘not at all’) to 6 (‘very much’); **Diet quality score ranges from 0 (worst) to 10 (best); §Fullness rating ranges from 1 (‘still hungry’) to 6 (‘very full’); iAUC: incremental area under the curve for 2-h postprandial glucose

Table 2 presents the percentage of meals consumed with different companions and activities, categorized by location. Most meals were consumed at home (60%), followed by hawker centers (local open-air food courts; 14%), the workplace (11%), and other restaurants (9%). Eating at fast food restaurants (2%) or a friend/relative’s home (1%) was less common. Meals were commonly consumed alone (34%) or with family members, including spouses (27%), children (15%), and other relatives (21%). In addition, a substantial proportion of meals was consumed with colleagues (9%) or friends (9%). Only 22% of meals were consumed without other activities. The most popular activities during meals were talking (42%) and leisure screen use (34%).

**Table 2:** Meal companion and activity during the meal according to meal location

	Meal companion						Activity during the meal				
	Alone	Spouse	Children	Other family	Friends	Colleagues	Nothing	Work	Leisure screen use	Talking	Leisure reading/writing
Meal location											
All locations	34%	27%	15%	21%	9%	9%	22%	8%	34%	42%	3%
Home (60%)	39%	30%	20%	26%	2%	0%	24%	5%	45%	33%	4%
Workplace (11%)	44%	1%	1%	1%	8%	47%	17%	35%	26%	35%	3%
Hawker center (14%)	28%	28%	10%	14%	18%	15%	26%	4%	17%	57%	2%
Fast food restaurant (2%)	22%	33%	18%	15%	23%	10%	15%	3%	17%	67%	2%
Other restaurant (9%)	9%	34%	13%	21%	33%	13%	11%	3%	10%	79%	1%
Friend/relative’s home (1%)	4%	34%	19%	57%	33%	1%	11%	1%	16%	82%	0%
*P*-value difference	< 0.001	< 0.001	< 0.001	< 0.001	< 0.001	< 0.001	< 0.001	< 0.001	< 0.001	< 0.001	< 0.001

*P*-values were obtained by comparing the null GEE model with the GEE model adjusted only for the context variable (set) of interest. The analysis was based on 20,629 meals. Meal companion and activity during the meal were collected using check-all-that-apply questions, where more than one response were allowed. Meal locations accounted for less than 1% of meals (e.g., on-the-go) were excluded

### Meal location and diet quality, fullness, and glucose levels

**Fig. 1:**
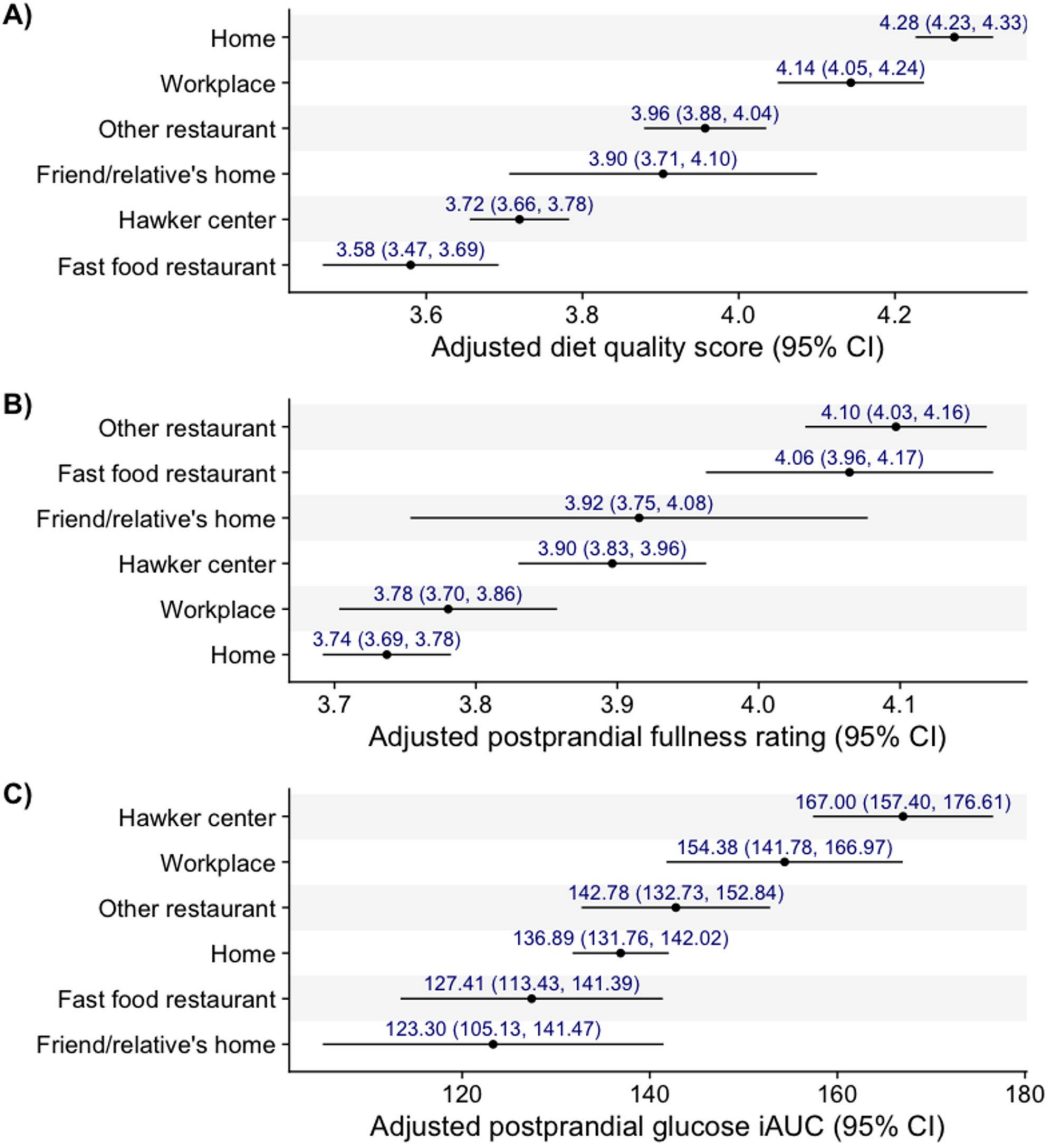
Adjusted diet quality, satiety, and postprandial glucose responses by meal location. **A** Diet quality. **B** Satiety. **C** Postprandial glucose responses. Estimates were adjusted for age, sex, ethnicity, education, smoking, alcohol consumption, working status, marital status, BMI, glycemic status, day of week, mealtime, meal companion, activity during the meal, and meal location

Supplementary Table 1 presents the diet quality score, perceived fullness rating, and postprandial glucose levels by meal location. Table 3 and Fig. 1 show these results after adjustment for potential confounders and other aspects of the eating context. Compared to eating at home, eating at all other locations was associated with significantly lower diet quality. Diet quality was lowest at fast-food restaurants (β: -0.70; 95% CI: -0.82, -0.57), followed by hawker centers (β: -0.56; CI: -0.63, -0.48), a friend or relative’s home (β: -0.37; CI: -0.57, -0.17), other restaurants (β: -0.32; CI: -0.41, -0.23), and the workplace (β: -0.13; CI: -0.23, -0.03)). Eating at other restaurants (β: 0.36; CI: 0.29, 0.43), fast food restaurants (β: 0.33; CI: 0.22, 0.43), hawker centers (β: 0.16; CI: 0.09, 0.22), and a friend or relative’s home (β: 0.18; CI: 0.02, 0.34) was associated with significantly greater postprandial fullness than eating at home. Postprandial glucose levels were significantly higher after eating at a hawker center (β: 30.11; CI: 20.11, 40.11) or at work (β: 17.48; CI: 4.34, 30.62) than after eating at home.

**Table 3:** Associations of eating context, including premeal psychophysiological states, with diet quality, satiety, and postprandial glucose response

	Diet quality score		Postprandial fullness rating		Postprandial glucose iAUC	
	β (95% CI)	*P*-value	β (95% CI)	*P*-value	β (95% CI)	*P*-value
Meal location (reference: home)						
Workplace	-0.13 (-0.23, -0.03)	0.010	0.04 (-0.04, 0.13)	0.300	17.48 (4.34, 30.62)	0.009
Hawker center	-0.56 (-0.63, -0.48)	< 0.001	0.16 (0.09, 0.22)	< 0.001	30.11 (20.11, 40.11)	< 0.001
Fast food restaurant	-0.70 (-0.82, -0.57)	< 0.001	0.33 (0.22, 0.43)	< 0.001	-9.48 (-23.93, 4.97)	0.198
Other restaurant	-0.32 (-0.41, -0.23)	< 0.001	0.36 (0.29, 0.43)	< 0.001	5.89 (-4.66, 16.43)	0.274
Friend/relative’s home	-0.37 (-0.57, -0.17)	< 0.001	0.18 (0.02, 0.34)	0.030	-13.59 (-31.88, 4.70)	0.145
Meal companion (reference: alone)						
Spouse	0.01 (-0.07, 0.10)	0.742	0.08 (0.02, 0.14)	0.011	0.05 (-8.68, 8.79)	0.991
Children	0.01 (-0.08, 0.10)	0.856	0.00 (-0.08, 0.09)	0.928	-5.07 (-15.17, 5.04)	0.325
Other family member	0.02 (-0.06, 0.09)	0.679	0.10 (0.03, 0.18)	0.006	-4.21 (-13.32, 4.89)	0.365
Friends	0.03 (-0.06, 0.11)	0.543	0.14 (0.06, 0.22)	< 0.001	-25.92 (-37.10, -14.75)	< 0.001
Colleagues	0.11 (0.01, 0.20)	0.027	0.10 (0.02, 0.19)	0.014	5.93 (-8.52, 20.39)	0.421
Activity during the meal (reference: only eating)						
Work	0.00 (-0.09, 0.09)	0.981	-0.20 (-0.28, -0.11)	< 0.001	-14.69 (-25.92, -3.47)	0.010
Leisure screen use	-0.02 (-0.09, 0.05)	0.628	0.02 (-0.03, 0.08)	0.412	5.03 (-2.50, 12.56)	0.190
Talking	0.03 (-0.05, 0.10)	0.471	0.02 (-0.03, 0.08)	0.427	2.86 (-4.95, 10.68)	0.473
Leisure reading/writing	0.18 (-0.05, 0.41)	0.120	-0.01 (-0.16, 0.13)	0.842	-5.83 (-19.80, 8.14)	0.414
Premeal psychological state*						
Stress	-0.01 (-0.03, 0.02)	0.710	0.02 (-0.01, 0.05)	0.216	-1.50 (-5.18, 2.19)	0.426
Hunger	-0.02 (-0.04, 0.01)	0.146	0.01 (-0.01, 0.03)	0.518	4.37 (1.53, 7.21)	0.003
Tiredness	-0.02 (-0.04, 0.01)	0.191	0.02 (-0.01, 0.04)	0.180	-0.62 (-4.18, 2.94)	0.732
Happiness	0.04 (0.01, 0.07)	0.018	0.06 (0.02, 0.09)	0.001	-0.74 (-4.76, 3.29)	0.720

The analysis for meal location, companions, and activities was based on 20,629 meals for diet quality, 20,582 for fullness, and 12,622 for glucose iAUC. The analysis for psychological state was based on 11,783 meals for diet quality, 11,767 for fullness, and 7161 for glucose iAUC. Estimates were adjusted for age, sex, ethnicity, education, smoking, alcohol consumption, working status, marital status, BMI, glycemic status, day of week, mealtime, meal companion, activity during the meal, and meal location. *Betas for premeal psychological state were expressed per unit increment in the scores and additionally adjusted for all premeal psychological state variables

To provide insight into potential reasons for differences in meal quality and postprandial glucose levels, we also examined the specific foods consumed at different locations (Supplementary Table 2). Foods consumed during meals in order of popularity were refined grains (69% of meals), vegetables (43%), chicken (24%), red meat (23%), seafood (21%), eggs (21%), whole grains (17%), legumes and nuts (17%), fruit (15%), deep-fried foods (9%), dairy (8%), and sweet desserts (7%). Foods consumed during meals at home were similar to the overall food consumption. In contrast, eating at a hawker center was associated with a higher intake of refined grains (83%) and red meat (31%), and a lower intake of whole grains (8%), fruit (7%), and dairy (2%). Eating at a fast-food restaurant was associated with a low consumption of refined (47%) and whole (10%) grains, vegetables (27%), fruit (4%), and legumes or nuts (4%), and the highest consumption of deep-fried foods (49%) and chicken (39%). Eating at another restaurant was associated with a high consumption of red meat (39%), seafood (39%), chicken (32%), and vegetables (50%), and a low consumption of fruit (9%) and whole grains (8%).

### Meal companions and activities and diet quality, fullness, and glucose levels

Meal companions and activities differed significantly by location (p-values < 0.001; Table 2). Meals at home were typically consumed alone or with a spouse, children, or other family members. In comparison, eating at another restaurant was less likely to be done alone, and meals at the workplace were usually consumed alone or with colleagues. Companions for meals at hawker centers and fast-food restaurants were more evenly divided between eating alone and with family members, friends, or colleagues. As expected, meals at a friend’s or relative’s home were usually consumed with family members or friends. In terms of activities, meals consumed at home were most commonly accompanied by leisure screen use, followed by talking or no other activity. At work, talking and working were the most common activities during the meal. For all other meal locations, the majority of the meals were consumed while talking.

Supplementary Table 1 shows diet quality, fullness, and glucose levels according to meal companions and activities. These associations were mostly explained by adjusting for potential confounders, including meal location (Table 3). Eating with colleagues was associated with slightly higher diet quality than eating alone. Additionally, eating with others, except for children, was associated with greater postprandial fullness than eating alone. Finally, eating with friends was associated with significantly lower postprandial glucose levels than eating alone. Regarding activities, working during meals was associated with lower post-meal fullness and glucose levels.

### Pre-meal psychological states

Unadjusted correlations between pre-meal psychological states (i.e., hunger, tiredness, stress, and happiness) and diet quality, fullness, and glucose levels are shown in Supplementary Tables 3, and the results after adjustment for confounders are shown in Table 3. Greater hunger was associated with higher postprandial glucose levels. Furthermore, greater happiness was associated with greater diet quality and postprandial fullness. This reflected modest positive correlations between happiness and intakes of vegetables (*r* = 0.08, *P* < 0.001) and fruit (*r* = 0.12, *P* < 0.001) (Supplementary Table 4). A sensitivity analysis with additional adjustments for psychological states for meal location, companion, and activity did not substantially change the results (Supplementary Table 5).

### Additional sensitivity analyses

Variance inflation factors assessing multicollinearity in the multivariable models ranged from 1.05 to 1.49, indicating minimal risk of multicollinearity. Sensitivity multivariable GEE models employing an exchangeable or autoregressive working correlation structure, despite a tendency toward being less conservative, yielded results that were largely consistent with the main analysis (Supplementary Table 6).

## Discussion

Our study provides insights into the impact of eating context on dietary quality and postprandial glucose levels for up to 20,629 free-living meals in an urban Asian setting. The location where meals were consumed had the strongest association with dietary quality and postprandial glucose levels. Meals consumed at home consistently had higher diet quality than those consumed at all out-of-home locations, with the worst dietary quality observed at hawker centers (local open-air food courts) and Western fast-food restaurants. In addition, greater happiness was associated with higher dietary quality, particularly a higher likelihood of consuming vegetables and fruits. The level of stress, hunger, or tiredness before meals was not associated with the diet quality of the meal after adjustment for other contextual variables. Fullness after meals was associated with who and where meals were consumed. Specifically, consuming meals with other adults, out-of-home (except for workplace meals), or with greater pre-meal happiness were associated with greater fullness after meals. These differences probably reflect differences in the amount of food consumed in different contexts. Furthermore, postprandial glucose levels after meals consumed at hawker centers and the workplace, but not at fast food or other restaurants, were significantly higher than those after meals consumed at home. Eating with friends was associated with lower postprandial glucose levels, whereas being hungrier before meals was associated with higher postprandial glucose levels. These increased glucose levels likely reflect greater food intake (as indicated by fullness) and the composition of meals.

Our observation of worse dietary quality for out-of-home meals is largely consistent with previous studies from other parts of the world. A systematic review by Bezerra et al. (2012) that included 28 studies, cross-sectional and prospective cohort studies from the United States, Europe, Australia, China, and Brazil, reported that frequently eating outside the home was generally positively associated with body weight and obesity risk [31]. A second systematic review by Lachat et al. in 2012 included 29 mostly cross-sectional studies from Europe, the United States, Russia, China, the Philippines, Vietnam, Kenya, and Australia [32]. They reported that eating outside of the home was generally associated with lower dietary quality [32]. Additionally, studies conducted in China and Korea showed that eating outside the home is associated with increased energy and sodium intake [33, 34]. A cross-sectional study by Shinozaki et al. employed EMA to examine associations between meal location and diet quality in Japan [11]. In this study, eating away from home was associated with higher diet quality, particularly in females. However, the authors pointed out that the majority of meals consumed outside the home were still prepared at home and consumed at work. Results from a previous mixed-methods study in Singapore suggest that, despite perceived meal unhealthiness, the cost of meals, restaurant proximity, and the variety of options were the main drivers of eating outside the home at hawker stalls and Western fast-food restaurants [17]. This provides context for why many choose to consume meals outside the home in Singapore despite reduced diet quality.

Prior studies have also examined mood in relation to diet quality. A systematic review of observational studies by Hill et al. found that stress was associated with reduced diet quality [10]. Fruit and vegetables have been associated with improved mental health indicators in a systematic review by Glabska et al. [9]. While our study did not investigate this direction of association, it did observe that happiness before meals was associated with higher diet quality of meals. Positive moods have been associated with healthier food choices because individuals are motivated to promote longevity and health [35].

Previous studies, primarily conducted in older adults, have shown that individuals who mostly consumed their meals alone were more likely to have low appetite, whereas those who consumed meals with others had higher energy intakes [36, 37]. Likewise, our current findings showed that postprandial fullness was greater when meals were consumed with other adults, including spouses, other family members, friends, and colleagues, compared with eating alone. Consumption of meals with friends and family may facilitate greater food intake through a variety of factors, such as mirroring your meal partners’ food volume, longer meal duration, and food sharing with companions [38].

We observed higher postprandial glucose levels for meals consumed at hawker centers, but not at fast-food restaurants, compared with home meals. A previous analysis of the COBRA study showed an association between reduced intake of refined grains, increased intake of protein-rich foods, and lower postprandial glucose levels [28]. This could explain the difference in postprandial glucose levels between hawker centers and fast-food restaurants, as refined grain consumption is higher and the consumption of protein-rich foods is lower at hawker centers. Greater food intake, as reflected in postprandial fullness, could contribute to higher glucose levels. However, this is unlikely to explain differences in postprandial glucose levels by eating location, as fullness was greater after eating at fast food and other restaurants than at workplaces and hawker centers. Consuming meals in the presence of friends was also associated with lower postprandial glucose levels [38]. This may reflect higher consumption of protein-rich foods or longer meal duration. In contrast, greater hunger before the meal was associated with higher postprandial glucose levels, likely reflecting greater food intake. To our knowledge, no prior research has linked elevated postprandial blood glucose levels to meal location, social company during mealtime, pre-meal psychological state, or activities during mealtime. However, a study by Kusuma et al. in India observed an increase in blood glucose levels and the risk of diabetes in areas with fast-food restaurants, using Geographic Information System (GIS) technology [6]. The limited prior research speaks to the utility of our current study.

Our study had several strengths. Data collection was primarily conducted through daily timed surveys, which enabled researchers to obtain real-time data on food consumption and eating context. By relying on real-time data collection rather than dietary recall, this method may reduce the likelihood of misclassification due to memory errors, which are common in recall-based data. Furthermore, our study featured continuous blood glucose monitoring. Limitations were also present in our study. The diet quality score used, while rooted in available literature regarding the health effects of different food groups and similar to the DASH score, was an unvalidated measure [3, 29, 39–41]. A second limitation was the lack of data on the portion sizes of foods consumed by participants. We decided to limit the collection of portion-size information to reduce participant burden, which may have contributed to the high EMA response rate in our study. In addition, our EMA questions did not distinguish between beverages consumed as part of a meal and those consumed outside of meal occasions. Therefore, we were unable to accurately include beverage consumption in our meal-based dietary quality score. Nevertheless, results were largely consistent from sensitivity analysis that incorporated proxy sugar-sweetened beverage consumption, using beverage intake reported in the same EMA survey as the meal, as an additional ‘unhealthy’ food component of dietary quality score (Supplementary Table 7). Third, given the observational study design, the reported associations may have been affected by residual confounding by factors that were not considered or imperfectly measured. Fourth, we conducted a large number of statistical tests, which increased the likelihood of chance findings. However, we primarily focused on associations with high levels of statistical significance. Finally, the study primarily consisted of ethnic Chinese individuals with an overrepresentation of persons with higher levels of education. Further research should confirm our findings in people with lower socioeconomic status and other urban Asian settings.

To conclude, our findings suggest that where you eat, who you eat with, and your mood can significantly affect diet quality, satiety, and postprandial glucose levels. Our study results provide direction for future policies and public health interventions to help improve dietary behaviors and highlight the impact of meal location on dietary quality. Our results highlight that, in addition to Western-style fast food restaurants, local food outlets in urban Asian settings can also be a source of meals with low dietary quality. Furthermore, these meals are often associated with strongly elevated postprandial glucose levels. Public health programs aimed at improving cooking skills and campaigns highlighting the health benefits of home-cooked meals could be used to encourage behavior change. Additionally, public health programs, such as Singapore’s Healthier Dining Program, can provide healthier food options while eating out [42]. When promoting dietary behavior change, employing multi-level health programming has the potential to yield high public health impacts to reduce chronic disease burden.

## Supplementary Information

Supplementary Material 1.

## Data Availability

The data used for this study are held by the Saw Swee Hock School of Public Health at the National University of Singapore. The data can be released to bona fide researchers upon reasonable requests and agreements via https://blog.nus.edu.sg/sphs/data-and-samples-request/. The data release must conform to the Personal Data Protection Act in Singapore.
