# Supplementary material for: Associations of eating context with dietary quality, satiety, and postprandial blood glucose in free-living Singaporean adults during 9 days of intensive digital phenotyping
Source: Int J Behav Nutr Phys Act. 2026 Mar 18;23:43. doi: 10.1186/s12966-026-01903-2 (PMC13123034; doi:10.1186/s12966-026-01903-2)
Supplement: Supplementary file 1 — Supplementary Material 1. [file 12966_2026_1903_MOESM1_ESM.docx]

**Supplementary Table 1: Diet quality, satiety, and postprandial glucose responses by eating context.**

|  | Diet quality score | Postprandial fullness | Postprandial glucose iAUC |
| --- | --- | --- | --- |
| **All meals** | 4.1 (1.4) | 3.8 (1.0) | 143.3 (137.5) |
| **Meal companion** | | | |
| Alone (34%) | 4.2 (1.4) | 3.7 (1.0) | 144.6 (140.2) |
| Spouse (27%) | 4.2 (1.5) | 3.9 (1.0) | 146.0 (137.7) |
| Children (15%) | 4.3 (1.4) | 3.8 (1.0) | 137.3 (131.7) |
| Other family (21%) | 4.1 (1.4) | 3.9 (1.0) | 130.9 (129.2) |
| Friends (9%) | 3.9 (1.3) | 4.1 (1.0) | 126.1 (131.3) |
| Colleagues (9%) | 4.0 (1.3) | 3.9 (1.0) | 170.7 (150.7) |
| **P-value for differences** | <0.001 | <0.001 | <0.001 |
| **Activity during the meal** | | | |
| Only eating (22%) | 4.2 (1.4) | 3.7 (1.0) | 148.3 (143.6) |
| Work (8%) | 4.0 (1.3) | 3.5 (1.1) | 134.0 (138.7) |
| Leisure screen use (34%) | 4.1 (1.4) | 3.8 (1.0) | 143.0 (136.7) |
| Talking (42%) | 4.1 (1.4) | 3.9 (1.0) | 141.9 (135.6) |
| Leisure reading/writing (3%) | 4.4 (1.4) | 3.6 (1.1) | 138.6 (126.0) |
| **P-value for differences** | <0.001 | <0.001 | 0.455 |
| **Meal location** | | | |
| Home (60%) | 4.3 (1.4) | 3.7 (1.0) | 134.4 (131.1) |
| Workplace (11%) | 4.1 (1.3) | 3.7 (1.0) | 159.4 (151.5) |
| Hawker center (14%) | 3.7 (1.2) | 3.9 (1.0) | 176.4 (149.3) |
| Fast food restaurant (2%) | 3.5 (1.2) | 4.2 (1.0) | 127.3 (127.8) |
| Other restaurant (9%) | 3.9 (1.3) | 4.2 (1.0) | 139.7 (132.5) |
| Friend/relative's home (1%) | 4.0 (1.4) | 4.1 (1.1) | 105.9 (120.1) |
| **P-value for differences** | <0.001 | <0.001 | <0.001 |

Data are mean (SD) or %. P-values were obtained by comparing the null GEE model with the GEE model only adjusted for the context variable. The analysis for diet quality scores was based on 20,629 meals, the analysis for postprandial fullness on 20,582 meals, and the analysis for postprandial glucose iAUC on 12,622 meals.

**Supplementary Table 2: Meal composition according to eating context**

|  | Meal composition | | | | | | | | | | | |
| --- | --- | --- | --- | --- | --- | --- | --- | --- | --- | --- | --- | --- |
|  | Refined grains | Whole grains | Seafood | Chicken | Red meat | Eggs | Dairy | Legumes or nuts | Vegetables | Fruit | Deep-fried food | Sweet desserts |
| **All meals** | 69% | 17% | 21% | 24% | 23% | 21% | 8% | 17% | 43% | 15% | 9% | 7% |
| **Meal location** | | | | | | | | | | | | |
| Home (60%) | 66% | 21% | 19% | 21% | 19% | 22% | 10% | 17% | 43% | 19% | 7% | 6% |
| Workplace (11%) | 71% | 18% | 15% | 30% | 15% | 20% | 7% | 15% | 41% | 10% | 8% | 5% |
| Hawker center (14%) | 83% | 8% | 22% | 24% | 31% | 25% | 2% | 16% | 43% | 7% | 7% | 4% |
| Fast food restaurant (2%) | 47% | 10% | 18% | 39% | 19% | 11% | 7% | 4% | 27% | 4% | 49% | 9% |
| Other restaurant (9%) | 73% | 8% | 39% | 32% | 39% | 22% | 7% | 20% | 50% | 9% | 16% | 14% |
| Friend/relative's home (1%) | 77% | 12% | 34% | 32% | 38% | 18% | 3% | 21% | 55% | 16% | 14% | 16% |
| **P-value for differences** | <0.001 | <0.001 | <0.001 | <0.001 | <0.001 | <0.001 | <0.001 | <0.001 | <0.001 | <0.001 | <0.001 | <0.001 |
| **Meal companion** | | | | | | | | | | | | |
| Alone (34%) | 65% | 21% | 14% | 20% | 16% | 21% | 10% | 16% | 37% | 14% | 6% | 5% |
| Spouse (27%) | 71% | 17% | 26% | 25% | 28% | 22% | 7% | 18% | 48% | 18% | 10% | 7% |
| Children (15%) | 72% | 17% | 27% | 28% | 28% | 23% | 9% | 18% | 52% | 20% | 10% | 7% |
| Other family (21%) | 73% | 15% | 28% | 27% | 27% | 22% | 6% | 18% | 47% | 15% | 12% | 9% |
| Friends (9%) | 73% | 9% | 30% | 32% | 30% | 21% | 6% | 18% | 45% | 11% | 13% | 11% |
| Colleagues (9%) | 74% | 13% | 21% | 35% | 22% | 22% | 4% | 17% | 46% | 9% | 10% | 6% |
| **P-value for differences** | <0.001 | <0.001 | <0.001 | <0.001 | <0.001 | 0.36 | <0.001 | <0.001 | <0.001 | <0.001 | <0.001 | <0.001 |
| **Activity during the meal** | | | | | | | | | | | | |
| Only eating (22%) | 66% | 20% | 18% | 19% | 18% | 21% | 8% | 17% | 39% | 16% | 5% | 4% |
| Work (8%) | 65% | 21% | 13% | 22% | 13% | 18% | 9% | 13% | 30% | 10% | 7% | 6% |
| Leisure screen use (34%) | 70% | 17% | 19% | 24% | 21% | 22% | 9% | 16% | 43% | 16% | 9% | 7% |
| Talking (42%) | 73% | 14% | 27% | 29% | 29% | 23% | 7% | 19% | 50% | 14% | 12% | 9% |
| Leisure reading/writing (3%) | 65% | 24% | 20% | 21% | 15% | 24% | 12% | 17% | 37% | 28% | 7% | 7% |
| **P-value for differences** | <0.001 | <0.001 | <0.001 | <0.001 | <0.001 | <0.001 | <0.001 | <0.001 | <0.001 | <0.001 | <0.001 | <0.001 |

Data are mean (SD) or %. The analysis was based on 20,629 meals. P-values were obtained by comparing the null GEE model with the GEE model adjusted only for the context variable. Meal composition was collected using check-all-that-apply questions, where more than one response were allowed. Meal locations accounted for less than 1% of meals (e.g., on-the-go) were excluded.

**Supplementary Table 3: Correlation coefficients between premeal psychophysiological states and diet quality, fullness, and glucose levels after the meal.**

| Premeal psychological state | Diet quality score | Postprandial fullness | Glucose iAUC |
| --- | --- | --- | --- |
| Stress | -0.08 (<0.001) | 0.02 (0.330) | -0.00 (0.821) |
| Hunger | -0.06 (<0.001) | 0.03 (0.090) | 0.04 (0.139) |
| Tiredness | -0.09 (<0.001) | 0.05 (0.006) | -0.00 (0.752) |
| Happiness | 0.10 (<0.001) | 0.04 (0.041) | 0.01 (0.826) |

Data are Pearson correlation coefficients (p-value). P-values were obtained using 10,000 bootstrapping samples (each bootstrapping sample represents one simulated dataset derived from the original meal dataset). The analysis was based on 11,783 meals for the diet quality score, 11,767 meals for postprandial fullness, and 7,161 meals for the postprandial glucose iAUC.

**Supplementary Table 4: Correlation coefficients between premeal psychophysiological states and meal composition**

| Premeal psychological state | Meal composition | | | | | | | | | | | |
| --- | --- | --- | --- | --- | --- | --- | --- | --- | --- | --- | --- | --- |
|  | Refined grains | Whole grains | Seafood | Chicken | Red meat | Eggs | Dairy | Legumes or nuts | Vegetables | Fruit | Deep-fried food | Sweet desserts |
| Stress | 0.02 (0.054) | -0.02 (0.119) | -0.03 (0.003) | 0.04 (0.003) | -0.02 (0.052) | -0.02 (0.162) | -0.04 (<0.001) | -0.04 (0.001) | -0.08 (<0.001) | -0.07 (<0.001) | -0.01 (0.199) | -0.01 (0.260) |
| Hunger | 0.03 (0.028) | -0.01 (0.352) | -0.03 (0.029) | 0.05 (<0.001) | 0.00 (0.961) | -0.02 (0.110) | -0.02 (0.073) | -0.02 (0.079) | -0.05 (<0.001) | -0.04 (0.003) | 0.03 (0.023) | -0.00 (0.924) |
| Tiredness | 0.03 (0.009) | -0.04 (0.001) | -0.00 (0.723) | 0.06 (<0.001) | -0.00 (0.927) | -0.01 (0.415) | -0.05 (<0.001) | -0.04 (0.001) | -0.07 (<0.001) | -0.07 (<0.001) | 0.02 (0.039) | -0.01 (0.515) |
| Happiness | -0.03 (0.044) | 0.03 (0.054) | 0.04 (0.004) | -0.01 (0.587) | 0.01 (0.583) | -0.00 (0.976) | 0.02 (0.063) | 0.03 (0.016) | 0.08 (<0.001) | 0.12 (<0.001) | 0.00 (0.757) | 0.01 (0.380) |

Data are r (p-value), where r refers to the point-biserial correlation coefficient. P-values were obtained using 10,000 bootstrap samples (each bootstrap sample is a simulated dataset from the original meal dataset). The data analysis was based on 11,783 meals.

**Supplementary Table 5: Associations of eating context with diet quality, satiety, and postprandial glucose response further adjusted for all premeal psychological state variables.**

|  | Diet quality score | | Postprandial fullness rating | | Postprandial glucose iAUC | |
| --- | --- | --- | --- | --- | --- | --- |
|  | β (95% CI) | P-value | β (95% CI) | P-value | β (95% CI) | P-value |
| **Meal location (reference: home)** | | | | | | |
| Workplace | -0.13 (-0.25, -0.01) | 0.038 | 0.00 (-0.10, 0.10) | 0.941 | 29.52 (12.44, 46.61) | 0.001 |
| Hawker center | -0.52 (-0.61, -0.43) | <0.001 | 0.12 (0.04, 0.19) | 0.002 | 34.35 (21.39, 47.31) | <0.001 |
| Fast food restaurant | -0.78 (-0.92, -0.63) | <0.001 | 0.32 (0.19, 0.44) | <0.001 | -7.68 (-25.73, 10.37) | 0.404 |
| Other restaurant | -0.37 (-0.47, -0.26) | <0.001 | 0.35 (0.27, 0.44) | <0.001 | 1.75 (-11.60, 15.09) | 0.798 |
| Friend/relative's home | -0.40 (-0.63, -0.17) | 0.001 | 0.12 (-0.08, 0.31) | 0.243 | -9.01 (-31.28, 13.25) | 0.427 |
| **Meal companion (reference: alone)** | | | | | | |
| Spouse | 0.05 (-0.05, 0.14) | 0.368 | 0.10 (0.02, 0.18) | 0.011 | -0.68 (-11.60, 10.24) | 0.902 |
| Children | -0.03 (-0.13, 0.08) | 0.584 | 0.01 (-0.08, 0.10) | 0.869 | -9.90 (-22.30, 2.49) | 0.117 |
| Other family member | 0.01 (-0.07, 0.10) | 0.813 | 0.08 (0.01, 0.16) | 0.034 | 1.20 (-10.47, 12.88) | 0.840 |
| Friends | 0.05 (-0.05, 0.15) | 0.300 | 0.11 (0.01, 0.20) | 0.024 | -25.00 (-38.68, -11.33) | <0.001 |
| Colleagues | 0.11 (-0.01, 0.23) | 0.067 | 0.11 (0.01, 0.21) | 0.030 | 2.30 (-15.09, 19.69) | 0.795 |
| **Activity during the meal (reference: only eating)** | | | | | | |
| Work | -0.06 (-0.18, 0.05) | 0.278 | -0.15 (-0.26, -0.04) | 0.007 | -16.37 (-30.34, -2.40) | 0.022 |
| Leisure screen use | 0.01 (-0.08, 0.09) | 0.897 | 0.02 (-0.05, 0.08) | 0.651 | 5.33 (-4.20, 14.86) | 0.273 |
| Talking | 0.01 (-0.08, 0.09) | 0.868 | 0.03 (-0.03, 0.10) | 0.345 | 2.21 (-7.39, 11.81) | 0.652 |
| Leisure reading/writing | 0.26 (-0.02, 0.54) | 0.066 | -0.08 (-0.27, 0.10) | 0.376 | -5.92 (-25.27, 13.43) | 0.549 |

Estimates were adjusted for age, sex, ethnicity, education, smoking, alcohol consumption, working status, marital status, BMI, glycemic status, day of week, mealtime, meal companion, activity during the meal, meal location, and premeal psychological state variables. The analysis was based on 11,783 meals for the diet quality score, 11,767 meals for postprandial fullness, and 7,161 meals for postprandial glucose.

**Supplementary Table 6: Associations of eating context, including premeal psychophysiological states, with diet quality, satiety, and postprandial glucose response using sensitivity multivariable GEE models employing an exchangeable working correlation structure and an autoregressive working correlation structure.**

| **Dependent variables** | **Exposure variables** | **GEE with exchangeable working correlation structure** | | **GEE with autoregressive working correlation structure** | |
| --- | --- | --- | --- | --- | --- |
|  |  | **β (95% CI)** | **P-value** | **β (95% CI)** | **P-value** |
| **Diet quality score** | **Meal location (reference: home)** | | | | |
|  | Workplace | -0.10 (-0.18, -0.01) | 0.021 | -0.12 (-0.21, -0.03) | 0.009 |
|  | Hawker center | -0.48 (-0.55, -0.41) | <0.001 | -0.50 (-0.57, -0.42) | <0.001 |
|  | Fast food restaurant | -0.64 (-0.76, -0.52) | <0.001 | -0.70 (-0.82, -0.57) | <0.001 |
|  | Other restaurant | -0.29 (-0.37, -0.20) | <0.001 | -0.32 (-0.41, -0.24) | <0.001 |
|  | Friend/relative's home | -0.28 (-0.46, -0.11) | 0.001 | -0.34 (-0.52, -0.15) | <0.001 |
|  | **Meal companion (reference: alone)** | | | | |
|  | Spouse | 0.01 (-0.05, 0.07) | 0.806 | 0.01 (-0.05, 0.08) | 0.685 |
|  | Children | 0.05 (-0.03, 0.12) | 0.207 | 0.05 (-0.03, 0.13) | 0.224 |
|  | Other family member | 0.07 (0.01, 0.12) | 0.021 | 0.06 (-0.01, 0.12) | 0.081 |
|  | Friends | 0.01 (-0.06, 0.09) | 0.716 | 0.03 (-0.05, 0.11) | 0.519 |
|  | Colleagues | 0.12 (0.04, 0.20) | 0.003 | 0.14 (0.06, 0.23) | 0.001 |
|  | **Activity during the meal (reference: only eating)** | | | | |
|  | Work | -0.02 (-0.09, 0.06) | 0.651 | -0.03 (-0.11, 0.05) | 0.487 |
|  | Leisure screen use | 0.01 (-0.04, 0.06) | 0.72 | 0.01 (-0.05, 0.06) | 0.821 |
|  | Talking | 0.01 (-0.04, 0.06) | 0.726 | 0.02 (-0.04, 0.08) | 0.504 |
|  | Leisure reading/writing | 0.04 (-0.08, 0.16) | 0.482 | 0.04 (-0.11, 0.20) | 0.559 |
|  | **(Premeal) Psychological state*** | | | | |
|  | Stress | -0.01 (-0.03, 0.02) | 0.584 | -0.01 (-0.03, 0.02) | 0.559 |
|  | Hunger | -0.02 (-0.04, 0.00) | 0.066 | -0.02 (-0.04, -0.00) | 0.046 |
|  | Tiredness | -0.00 (-0.03, 0.02) | 0.726 | -0.01 (-0.03, 0.02) | 0.545 |
|  | Happiness | 0.02 (-0.01, 0.04) | 0.186 | 0.03 (0.00, 0.06) | 0.043 |
| **Postprandial fullness rating** | **Meal location (reference: home)** | | | | |
|  | Workplace | 0.01 (-0.05, 0.07) | 0.742 | 0.02 (-0.05, 0.08) | 0.604 |
|  | Hawker center | 0.22 (0.18, 0.26) | <0.001 | 0.24 (0.19, 0.29) | <0.001 |
|  | Fast food restaurant | 0.30 (0.23, 0.38) | <0.001 | 0.33 (0.24, 0.42) | <0.001 |
|  | Other restaurant | 0.38 (0.33, 0.43) | <0.001 | 0.39 (0.33, 0.45) | <0.001 |
|  | Friend/relative's home | 0.18 (0.05, 0.31) | 0.007 | 0.17 (0.00, 0.33) | 0.048 |
|  | **Meal companion (reference: alone)** | | | | |
|  | Spouse | 0.08 (0.04, 0.11) | <0.001 | 0.07 (0.02, 0.11) | 0.003 |
|  | Children | 0.04 (-0.00, 0.09) | 0.079 | 0.05 (-0.01, 0.11) | 0.082 |
|  | Other family member | 0.12 (0.08, 0.16) | <0.001 | 0.14 (0.09, 0.19) | <0.001 |
|  | Friends | 0.09 (0.03, 0.14) | 0.002 | 0.08 (0.02, 0.15) | 0.011 |
|  | Colleagues | 0.12 (0.06, 0.18) | <0.001 | 0.13 (0.06, 0.19) | <0.001 |
|  | **Activity during the meal (reference: only eating)** | | | | |
|  | Work | -0.13 (-0.19, -0.08) | <0.001 | -0.13 (-0.20, -0.07) | <0.001 |
|  | Leisure screen use | 0.08 (0.05, 0.11) | <0.001 | 0.10 (0.06, 0.14) | <0.001 |
|  | Talking | 0.08 (0.05, 0.12) | <0.001 | 0.12 (0.07, 0.16) | <0.001 |
|  | Leisure reading/writing | 0.04 (-0.04, 0.12) | 0.365 | 0.04 (-0.05, 0.13) | 0.405 |
|  | **(Premeal) Psychological state*** | | | | |
|  | Stress | 0.00 (-0.02, 0.02) | 0.947 | -0.02 (-0.04, 0.01) | 0.194 |
|  | Hunger | 0.02 (0.01, 0.03) | 0.005 | 0.02 (0.01, 0.04) | 0.001 |
|  | Tiredness | 0.01 (-0.01, 0.02) | 0.339 | 0.01 (-0.01, 0.03) | 0.392 |
|  | Happiness | 0.03 (0.01, 0.05) | 0.002 | 0.02 (-0.01, 0.04) | 0.194 |
| **Postprandial glucose iAUC** | **Meal location (reference: home)** | | | | |
|  | Workplace | 13.89 (3.33, 24.44) | 0.01 | 14.94 (3.51, 26.36) | 0.01 |
|  | Hawker center | 30.21 (21.99, 38.42) | <0.001 | 32.82 (23.76, 41.88) | <0.001 |
|  | Fast food restaurant | -7.49 (-20.73, 5.75) | 0.267 | -1.35 (-15.73, 13.02) | 0.853 |
|  | Other restaurant | 6.83 (-2.61, 16.27) | 0.156 | 9.55 (-0.87, 19.97) | 0.072 |
|  | Friend/relative's home | -11.35 (-29.35, 6.65) | 0.217 | -10.97 (-31.99, 10.06) | 0.307 |
|  | **Meal companion (reference: alone)** | | | | |
|  | Spouse | -5.57 (-13.22, 2.07) | 0.153 | -3.71 (-12.26, 4.84) | 0.396 |
|  | Children | -10.29 (-18.55, -2.02) | 0.015 | -9.94 (-19.03, -0.85) | 0.032 |
|  | Other family member | -6.55 (-13.32, 0.23) | 0.058 | -6.11 (-13.76, 1.55) | 0.118 |
|  | Friends | -21.78 (-31.00, -12.56) | <0.001 | -23.06 (-33.87, -12.25) | <0.001 |
|  | Colleagues | 9.34 (-2.75, 21.42) | 0.13 | 4.97 (-8.12, 18.05) | 0.457 |
|  | **Activity during the meal (reference: only eating)** | | | | |
|  | Work | -15.91 (-25.29, -6.52) | 0.001 | -18.97 (-29.13, -8.82) | <0.001 |
|  | Leisure screen use | 7.15 (1.15, 13.14) | 0.019 | 5.19 (-1.33, 11.71) | 0.119 |
|  | Talking | 0.28 (-5.96, 6.53) | 0.929 | 0.46 (-6.50, 7.43) | 0.896 |
|  | Leisure reading/writing | -4.27 (-17.13, 8.60) | 0.516 | -4.53 (-17.78, 8.72) | 0.503 |
|  | **(Premeal) Psychological state*** | | | | |
|  | Stress | -0.73 (-3.73, 2.27) | 0.632 | 0.13 (-3.08, 3.34) | 0.938 |
|  | Hunger | 6.59 (4.16, 9.02) | <0.001 | 6.21 (3.57, 8.85) | <0.001 |
|  | Tiredness | -0.45 (-3.24, 2.35) | 0.754 | -0.45 (-3.58, 2.67) | 0.776 |
|  | Happiness | -2.73 (-5.80, 0.35) | 0.083 | -1.66 (-5.07, 1.75) | 0.34 |

The analysis for meal location, companions, and activities was based on 20,629 meals for diet quality, 20,582 for fullness, and 12,622 for glucose iAUC. The analysis for psychological state was based on 11,783 meals for diet quality, 11,767 for fullness, and 7,161 for glucose iAUC. Estimates were adjusted for age, sex, ethnicity, education, smoking, alcohol consumption, working status, marital status, BMI, glycemic status, day of week, mealtime, meal companion, activity during the meal, and meal location. *Betas for premeal psychological state were expressed per unit increment in the scores and additionally adjusted for all premeal psychological state variables.

**Supplementary Table 7: Associations of eating context with sensitivity diet quality that additionally integrated proxy sugar-sweetened beverage consumption (score range: 0-11)**

|  | **β (95% CI)** | **P-value** |
| --- | --- | --- |
| **Meal location (reference: home)** | | |
| Workplace | -0.17 (-0.27, -0.06) | 0.002 |
| Hawker center | -0.62 (-0.70, -0.54) | <0.001 |
| Fast food restaurant | -0.78 (-0.91, -0.65) | <0.001 |
| Other restaurant | -0.37 (-0.46, -0.28) | <0.001 |
| Friend/relative's home | -0.51 (-0.73, -0.28) | <0.001 |
| **Meal companion (reference: alone)** | | |
| Spouse | 0.00 (-0.08, 0.09) | 0.932 |
| Children | -0.02 (-0.11, 0.08) | 0.743 |
| Other family member | 0.01 (-0.07, 0.09) | 0.778 |
| Friends | 0.02 (-0.07, 0.11) | 0.623 |
| Colleagues | 0.09 (-0.01, 0.19) | 0.076 |
| **Activity during the meal (reference: only eating)** | | |
| Work | -0.02 (-0.11, 0.08) | 0.718 |
| Leisure screen use | -0.08 (-0.15, -0.00) | 0.043 |
| Talking | -0.03 (-0.10, 0.04) | 0.415 |
| Leisure reading/writing | 0.10 (-0.09, 0.30) | 0.308 |
| **(Premeal) Psychological state*** | | |
| Stress | -0.00 (-0.03, 0.03) | 0.977 |
| Hunger | -0.03 (-0.05, -0.00) | 0.018 |
| Tiredness | -0.02 (-0.05, 0.00) | 0.097 |
| Happiness | 0.03 (-0.00, 0.06) | 0.06 |

The analysis for meal location, companions, and activities was based on 20,629 meals for the sensitivity diet quality score that integrated proxy sugar-sweetened beverage consumption as an additional ‘unhealthy’ food component (value range: 0-11). The analysis for psychological state was based on 11,783 meals. Estimates were adjusted for age, sex, ethnicity, education, smoking, alcohol consumption, working status, marital status, BMI, glycemic status, day of week, mealtime, meal companion, activity during the meal, and meal location. *Betas for premeal psychological state were expressed per unit increment in the scores and additionally adjusted for all premeal psychological state variables
